# 

**Published:** 1857-01

**Authors:** 


### RECEIPTS ON SUBSCRIPTIONS,

UP TO DEC. 30TH, 1856.

**ILLINOIS.**

| | | |
|---|---|---|
| Dr. H. J. Vanwinkle, | Vol. 6, | $2 00 |
| " W. R. Griswold, | " 5, | 2 00 |
| " Bennett, | " 5, | 2 00 |
| " F. Seely, | Vol. 4 & 5, | 4 00 |
| " W. B. Hart, | Vol. 5, | 2 00 |
| " E. Harwood, | " 5, | 2 00 |
| " York & McClure, | " 5, | 2 00 |
| " W. R. Fox, | " 6, | 2 00 |
| " T. G. Vandever, | " 5, | 2 00 |
| " N. English, | Vol. 4 & 5, | 4 00 |
| " E. C. Ellett, | Vol. 6, | 2 00 |
| " Bray, | " 6, | 2 00 |
| " John Walker, | " 6, | 2 00 |

**INDIANA.**

| | | |
|---|---|---|
| Dr. A. McD. Thomas, | Vol. 6, | $2 00 |
| " John Newton, | Vol. 5 & 6, | 4 00 |
| " A. W. Adair, | Vol. 6, | 2 00 |

**INDIANA—Continued.**

| | | |
|---|---|---|
| Dr. Sam'l Ross, | Vol. 5, | 2 00 |
| " R. A. Perkins, | " 5, | 2 00 |
| " John Pickard, | " 6, | 2 00 |

**MICHIGAN.**

| | | |
|---|---|---|
| Dr. S. H. Whittlesey, | Vol. 5, | $2 00 |
| " J. Tripp, | " 5, | 2 00 |

**WISCONSIN.**

| | | |
|---|---|---|
| Dr. J. W. Perry, | Vol. 5, | $2 00 |
| " J. N. Millar, | " 5, | 2 00 |
| " B. O. Reynolds, | " 5, | 2 00 |
| " A. Clark, | Vol. 4 & 5, | 4 00 |

**IOWA.**

| | | |
|---|---|---|
| Dr. Ephraim Clifford, | Vol. 5, | $2 00 |
| " R. W. Hall, | Vol. 5 & 6, | 4 00 |

